# Comparison of risk factors for dementia in an urban vs rural areas in an elderly population cohort based in Hyderabad, India

**DOI:** 10.1002/alz70860_100057

**Published:** 2025-12-23

**Authors:** Vidyani Suryadevara, Vineela Vepakomma, Bhavana Pulivarthi, Kiran K Angadi, Francesca Mangialasche, Miia Kivipelto, Bhanuprakash Reddy G

**Affiliations:** ^1^ Stanford University, Stanford, CA, USA; ^2^ National Institute of Nutrition, Hyderabad, Telangana, India; ^3^ National Institute of Nuitrition, Hyderabad, Telangana, India; ^4^ Division of Clinical Geriatrics, Center for Alzheimer Research, Department of Neurobiology, Care Sciences and Society, Karolinska Institutet, Stockholm, Sweden; ^5^ Karolinska Institutet, Stockholm, Sweden

## Abstract

**Background:**

Dementia continues to be a growing problem in India, one of the most diverse countries in the world. Dementia is a multi‐factorial disorder driven by genetic predisposition and environmental influences, such as nutrition, resulting from lifelong interactions between protective and risk determinants. Modifying or controlling lifestyle‐related risk factors through targeted interventions may significantly reduce the risk of developing dementia. In this study, we deployed the most widely used Cardiovascular Risk Factors, Aging, and Incidence of Dementia (CAIDE) risk score for predicting the likelihood of onset of dementia in urban vs rural areas.

**Methods:**

This study performed at National Institute of Nutrition (NIN), India is community‐based cross‐sectional study in healthy individuals residing in and around Hyderabad, Telangana, in Southern India. The study included 471 individuals with an equal number of males and females with ages ranging 50‐90. This is a collaborative work with investigators form NIN, Worldwide FINGER and Stanford University. Among the various information collected from these individuals, the variables necessary to calculate CAIDE score included age, gender, level of education, BMI, physical activity, systolic blood pressure, and total cholesterol.

**Results:**

CAIDE score initially developed by the worldwide FINGER team was optimized to account for parameters specific to Indian population. In this cohort, we considered Individuals with a CAIDE score ≥9.0 to be at risk for onset of dementia. Among the study participants, 40% had a predicted risk of dementia, with a CAIDE score of ≥9.0 (*n* = 189). The proportion of individuals at risk (CAIDE score ≥9.0) was higher in rural areas (56%) compared to urban areas (28%). One striking difference in the variables between urban and rural cohorts was education levels.

**Conclusion:**

The study revealed a higher prevalence of risk for dementia in urban cohorts compared to the rural cohorts. Furthermore, the findings highlight the need for curating region‐specific lifestyle interventions to prevent the onset of dementia, given the vast diversity across disciplines.

